# Evaluation of UV-C Radiation Efficiency in the Decontamination of Inanimate Surfaces and Personal Protective Equipment Contaminated with Phage ϕ6

**DOI:** 10.3390/microorganisms10030593

**Published:** 2022-03-09

**Authors:** Maria Bartolomeu, Márcia Braz, Pedro Costa, João Duarte, Carla Pereira, Adelaide Almeida

**Affiliations:** Department of Biology and CESAM, Campus Universitário de Santiago, University of Aveiro, 3810-193 Aveiro, Portugal; maria.bartolomeu@ua.pt (M.B.); marciabraz96@ua.pt (M.B.); pedrommrscosta@ua.pt (P.C.); j.macedoduarte@ua.pt (J.D.)

**Keywords:** SARS-CoV-2 surrogate, phage ϕ6, UV-C light, disinfection, surfaces, personal protective equipment

## Abstract

To help halt the global spread of the severe acute respiratory syndrome coronavirus 2 (SARS-CoV-2), appropriate disinfection techniques are required. Over the last years, the interest in Ultraviolet-C (UV-C) radiation as a method to disinfect inanimate surfaces and personal protective equipment (PPE) has increased, mainly to efficiently disinfect and prevent SARS-CoV-2 from spreading and allow for the safe reuse of said equipment. The bacteriophage ϕ6 (or simply phage ϕ6) is an RNA virus with a phospholipid envelope and is commonly used in environmental studies as a surrogate for human RNA-enveloped viruses, including SARS-CoV-2. The present study investigated the use of two new UV irradiation systems ((2)2.4W and (8)5.5W)) constituted by conventional mercury UV-C lamps with a strong emission peak at ~254 nm to potentially inactivate phage ϕ6 on different surfaces (glass, plastic, stainless steel, and wood) and personal protective equipment, PPE, (surgical and filtering facepiece 2, FFP2, masks, a clear acetate visor, and disposable protective clothing). The results showed that both UV-C systems were effective in inactivating phage ϕ6, but the UV-C sterilizing chamber (8)5.5W had the best disinfection performance on the tested surfaces. The inactivation effectiveness is material-dependent on all surfaces, reaching the detection limit of the method at different times (between 60 and 240 s of irradiation). The glass surface needed less time to reduce the virus (30 s) when compared with plastic, stainless, and wood surfaces (60 s). The virus inactivation was more effective in the disposable surgical and FFP2 masks (60 and 120 s, respectively) than in the disposable vest and clear acetate visor (240 s). Overall, this study suggests that UV-C lamps with peak emission at ~254 nm could provide rapid, efficient, and sustainable sanitization procedures to different materials and surfaces. However, dosage and irradiation time are important parameters to be considered during their implementation as a tool in the fight against human coronaviruses, namely against SARS-CoV-2.

## 1. Introduction

The COVID-19 pandemic disease caused by the SARS-CoV-2 is a serious health problem worldwide [1]. The respiratory virus SARS-CoV-2 is shed in high concentrations in respiratory secretions and is mainly transmitted through respiratory droplets and close contact with infected people [2]. Furthermore, this virus can also spread through nasal, oral, and ocular mucus-contaminated surfaces [3]. The importance of contaminated surfaces and fomites in the transmission is uncertain [4]. However, some studies have demonstrated that SARS-CoV-2 can survive on static surfaces such as metal, glass, or plastic for several days [5,6]. Chin et al. (2020) found that it remained viable for up to 1 day on cloth and wood, up to 2 days on glass, 4 days on stainless steel and plastic, and up to 7 days on the outer layer of a medical mask [5]. Another study found that the virus survived 4 h on copper, 24 h on cardboard, and up to 72 h on plastic and stainless steel [6]. Gidari et al. (2021) observed that SARS-CoV-2 remained viable on plastic and glass for 120 h and stainless steel for 72 h [7]. In fact, Gonçalves et al. (2021) reported that the highest rate of SARS-CoV-2 was found in COVID-19 isolation wards, followed by a single study in a diagnostic laboratory, public transport systems, and long-term care facilities [8]. Recently, Kampf et al. (2020) observed that the detection rate of SARS-CoV was variable on inanimate surfaces (0–75%) of intensive care units, in isolation rooms (1.4–100%), and in general wards (0–61%) [9]. The coronavirus concentrations per swab were 4.4–5.2 log_10_ on intensive care units and 2.8–4.0 log_10_ on general wards [10]. In another study, Lv et al. (2020) reported that the materials/surfaces with the highest density of SARS-CoV-2 nucleic acid were outer gloves (37.4 copies/cm^2^), followed by door handles (26.25 copies/cm^2^), goggles (22.16 copies/cm^2^), and the outer cover and the inner wall of a high-speed centrifuge (19.95 and 14.70 copies/cm^2^, respectively) [11]. The level of contamination on surfaces and PPE is influenced by several factors that include the status of COVID-19 patients in the vicinity of the sampling areas, cleaning and disinfection, environmental factors (such as temperature, pH, and humidity), sampling procedures, and detection methods [5,8,12,13,14,15].

With the rapid increase in COVID-19 cases worldwide, strategies were implemented to limit the spread of SARS-CoV-2, including increased use of PPE and cleaning and disinfection procedures [16]. Such procedures may include heat sterilization, chemical disinfectants, filtration, and UV irradiation [1,7]. However, UV radiation disinfection is the one that offers the most advantages because it can be performed automatically and can be employed to efficiently control microbial growth in any medium and disinfect surfaces, liquids, air, and rooms; it is also very energy efficient [1,16]. In particular, UV-C light at wavelengths of approximately 254 nm exerts bactericidal and virucidal effects, and it is widely used to disinfect enclosed spaces [17].

UV light can directly affect viruses by modifying their genetic material (either DNA or RNA) and/or damaging their proteins/lipids, but inactivation is dominated by nucleic acid damage [1,18,19]. Absorbed UV-C light damages the virus nucleic acid, inducing the photochemical fusion of two adjacent pyrimidines into covalent-linked dimers, namely uracil/cytosine dimers in RNA and thymine/cytosine dimers in DNA [1,18,19]. Other mechanisms of UV-light inactivation include RNA-protein cross-linking [20] and site-specific damage to RNA or proteins through energy transfer between the two molecules [21]. This then leads to the cell’s inability to replicate and jeopardises its survival [1,22]. Nonetheless, each microbe has a different susceptibility to the UV wavelength and this will alter the effectiveness of the UV disinfection mechanism [1]. This susceptibility varies widely with the type of nucleic acids (DNA or RNA) and, as it happens in most viruses, with its genome structure (single- or double-stranded) [23]. Since lethal UV irradiation photoproducts are commonly thymine dimers, RNA viruses, such as the SARS-CoV-2 virus, are more resistant to UV radiation damage than DNA viruses [24,25]. However, capsid proteins, which protect the nucleic acids [26,27], are also easily disrupted (degraded and aggregated) by UV irradiation, leading to structural changes in the virus, impacting the virus’s ability to infect [28]. UV exposure also causes an increase in methyl groups associated with lipids, lipid oxidation, and also leads to alterations in lipid composition of viral lipidic envelopes [29] present in enveloped viruses, such as in the SARS-CoV-2 virus, surrounding the protein capsid. This makes the SARS-CoV-2 highly susceptible to UV-C irradiation and, as different studies have demonstrated, can lead to its rapid inactivation on surfaces and supplies [30,31,32,33,34]. The UV-C irradiation efficacy is substantially reduced by the increasing distance between the light source and the target, the surface’s matrix, and light reflection and scattering [35,36]. Therefore, the ideal fomites for UV-C decontamination should be non-porous and smooth, allowing them to be placed under the bulbs for short exposure cycles [36].

This study aimed to evaluate the efficiency of two UV irradiation systems on in-animate surfaces (plastic, glass, stainless steel, and wood and PPE (surgical and FFP2 masks, clear acetate visor, and disposable protective clothing)) contaminated with phage Φ6, used as SARS-CoV-2 surrogate, as additional help in the fight against the COVID-19 pandemic. Given the difficulty (pathogenicity, genetic mutations frequency, biosafety level 3/4) in working with highly infectious human viruses, namely coronaviruses, many researchers have considered the potential of phages, viruses that only infect bacteria, as models to measure the survival, transfer, and removal of human viruses [37,38,39]. Phage ϕ6 is an enveloped RNA virus that has been suggested as a good surrogate for the study of enveloped RNA viruses [40,41,42,43] such as SARS-CoV-2 [44]. Similar to SARS-CoV-2, phage ϕ6 has a lipid membrane, spike proteins, similar size (~80–100 nm) [44] and has an RNA genome (SARS-CoV-2 is a non-segmented single-stranded positive-sense RNA, with a length of ~30 kb [45] and phage ϕ6 is a three-part, segmented, double-stranded RNA genome, with ~13.5 kb in length [46]).

## 2. Materials and Methods

### 2.1. Bacterial Strain and Growth Conditions

Phage ϕ6 bacterial host strain, *Pseudomonas syringae* pv. *syringae* (DSM 21482), was acquired from Leibniz-Institute DSMZ—Deutsche Sammlung von Mikroorganismen und Zellkulturen GmmH (Braunschweig, Germany) [47]. The bacterial strain was stored at −80 °C in 10% glycerol. Before each assay, a stock culture of the bacteria was aseptically inoculated in 30 mL of Tryptic Soy Broth (TSB; Liofilchem, Roseto degli Abruzzi, Italy) and was grown for 18 h at 25 °C at 120 rpm stirring. An aliquot (300 µL) of this bacterial culture was transferred into a new fresh TSB medium and grown overnight at 25 °C to reach an absorbance at 600 nm of 0.8 (HaloDB-20; DynamicaScientific, Livingston, UK), corresponding approximately to 10^9^ cells per mL.

### 2.2. Preparation of Virus Suspension

Phage ϕ6 (DSM 21518) was used in this study as a model of SARS-CoV-2 and was acquired from Leibniz-Institute DSMZ—Deutsche Sammlung von Mikroorganismen und Zellkulturen GmmH (Braunschweig, Germany) [47]. Phage ϕ6 is an RNA phage that belongs to the *Cystoviridae* family [48,49]. Phage suspensions were prepared from a phage stock previously prepared in SM buffer [0.1 M NaCl (Sigma, St. Louis, MO, USA), 8 mM MgSO_4_ (Sigma, St. Louis, MO, USA), 20 mM Tris-HCl (Sigma, St. Louis, MO, USA), 2% (*w*/*v*) gelatin, pH 7.5)] using *P. syringae* pv. *syringae* as the host and according to a previous study of our research group [47]. The phage suspension was stored at 4 °C until use and the titer was determined through the double-layer agar method [50]. Successive dilutions of the phage suspension were performed in phosphate-buffered saline [PBS; 137 mM NaCl (Sigma, St. Louis, MO, USA), 2.7 mM KCl (Sigma, St. Louis, MO, USA), 8.1 mM Na_2_HPO_4_·2H_2_O, 1.76 mM KH_2_PO_4_ (Sigma, St. Louis, MO, USA), pH 7.4], and 500 µL of each dilution was added to 200 µL of fresh *P. syringae* pv. *syringae* culture, mixed with 5 mL of TSB 0.6% top agar layer [30 g/L TSB (Liofilchem, Roseto degli Abruzzi, Italy), 6 g/L agar (Liofilchem, Roseto degli Abruzzi, Italy), 0.05 g/L CaCl_2_ (Sigma, St. Louis, MO, USA), 0.12 g/L MgSO_4_ (Sigma, St. Louis, MO, USA), pH 7.4)] and poured over a Tryptic Soy Agar (TSA; Liofilchem, Roseto degli Abruzzi, Italy) plate. The plates were incubated at 25 °C for 18 h and the results were expressed as plaque-forming units per millilitre (PFU/mL).

### 2.3. UV-C Light System, Lamps Characteristics, and Irradiation Systems

**Figure 1:**
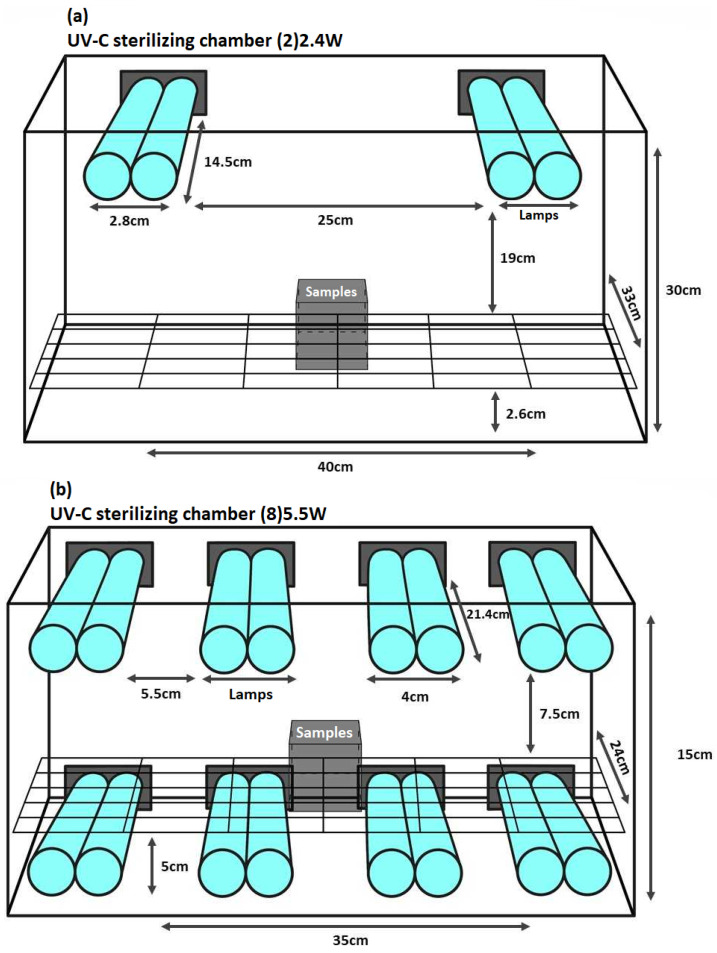
UV-C light disinfection systems. (**a**) UV-C sterilizing chamber (2)2.4W; the samples were placed approximately 20 cm from the two lamps and (**b**) UV-C sterilizing chamber (8)5.5W; the samples were placed approximately 7 cm from the top four lamps and approximately 2 cm from the bottom four lamps.

The UV-C sterilizing chamber (2)2.4W (Climar lighting) included two compact fluorescent germicidal UV-C lamps [9 W nominal power, 2-pin G23 base, twin-tube lamp with dimensions of 14.5 cm length and 2.8 cm diameter, operating on 120–277 V AC @ 50/60 Hz, (FRL-6-1X-G23-9W-UVC, Larson Electronics, Kemp, TX, USA)] providing a 2.4W UV-C output at a UV wavelength of 253.7 nm (Figure 1a). The UV-C lamps have low mercury content, are long-lasting due to the specific coating, and are ozone-free. The samples were placed approximately 20 cm from the two lamps (Figure 1a), with an average irradiance between 1.0–2.5 mW/cm^2^.

The UV-C sterilizing chamber (8)5.5W light disinfection system included eight UV-C germicidal lamps [18 W nominal power, 2G11 base, 21.4 cm length and 4.0 cm diameter, 58 V (Puritec HNS L 18 W 2G11, Osram, Garching, Germany)] providing a 5.5W UV-C output between 200–280 nm and a prevalent wavelength of 254 nm. The UV-C lamps have low mercury content, are long-lasting due to the specific coating, and are ozone-free. The samples were placed approximately 7 cm from the top four lamps and approximately 2 cm from the bottom four lamps (Figure 1b), with an average irradiance of 4.0 mW/cm^2^.

The average irradiance at samples’ level was measured with a laser power and energy meter FieldMaxII-TOP combined with a high-sensitivity thermopile sensor PS19Q (Coherent, Santa Clara, CA, USA).

The interior walls of both systems are lined with aluminium sheets, a highly reflective material.

### 2.4. Inanimate Surfaces and Personal Protective Equipment Preparation

The inanimate surfaces selected were plastic (polystyrene Petri dishes were used, Ø 90 mm, A = 63.60 cm^2^), glass (borosilicate Petri dishes were used, Ø 90 mm, A = 63.60 cm^2^), metal (stainless steel 304 L, A = 18.75 cm^2^), and wood (white-melamine faced chipboard sheets, A = 9.61 cm^2^). All materials were sterilized before use: polystyrene Petri dishes were purchased already sterilized by gamma rays; borosilicate Petri dishes and stainless-steel cuts were sterilized by autoclave; and wood pieces were disinfected with ethanol (70%) and sterilized with prolonged UV-C cycles of 2 min.

The PPE used in this work were surgical and FFP2 masks, clear acetate visor, and medical disposable protective clothing. Before each test, the PPE materials were cut into pieces (A = 6.0 cm^2^ to surgical and FFP2 masks, and medical disposable protective clothing; A = 63.60 cm^2^ to clear acetate visor). The PPE materials were sterilized before use by UV radiation for 2 min. After sterilization, the PPE pieces were aseptically placed in sterile Petri dishes.

### 2.5. UV-C Irradiation Assays

Inanimate surfaces and PPE were artificially contaminated with phage ϕ6 at a concentration of approximately 10^8^ PFU/mL. Surfaces and PPE were inoculated with ten droplets containing 10 μL of the prepared inoculum, which retained their shape. Surfaces and PPE were exposed to UV-C radiation while the inoculum was still wet. Surfaces (glass, plastic, stainless steel, and wood) were exposed to both UV-C sterilizing systems. The PPE materials were only exposed to the UV-C sterilizing chamber (8)5.5W (selected according to the results of the tests on surfaces). Non-irradiated controls were also included in the different experiments. After exposure, aliquots of the surface test samples and controls were collected in 1 mL of PBS at time 0 and after 30, 60, and 120 s of irradiation. For the PPE, aliquots were collected at time 0 and after 30, 60, 120, 180, 240, 300, and 360 s of irradiation. Successive dilutions of the treated samples and controls were performed in sterile PBS and plated by the drop-plate method (5 µL) in double-layered agar plates [50]. The plates were incubated at 25 °C for 12 h. The results were expressed in log PFU/mL as a function of irradiation time, in seconds. The detection limit of this method was 200 PFU/mL. Three independent experiments were performed for each condition and in each trial, three replicates were included.

### 2.6. Statistical Analysis

Statistical analysis of the data was performed using the GraphPad Prism 7.04 software (San Diego, CA, USA). After verifying the normal distribution of the data, the significance of viral concentrations between treatments, and throughout the trials, were evaluated by two-way ANOVA variance analysis. Tukey’s multiple comparison test was used for pairwise comparison of means. For different treatments, the significance of the differences was evaluated by comparing the results obtained in the test samples with each other and with the results obtained in the controls, for different times. A *p*-value < 0.05 was considered significant. Three independent experiments with three replicates for each were performed.

## 3. Results

### 3.1. UV-C Irradiation Assays

#### 3.1.1. Inanimate Surfaces

**Figure 2:**
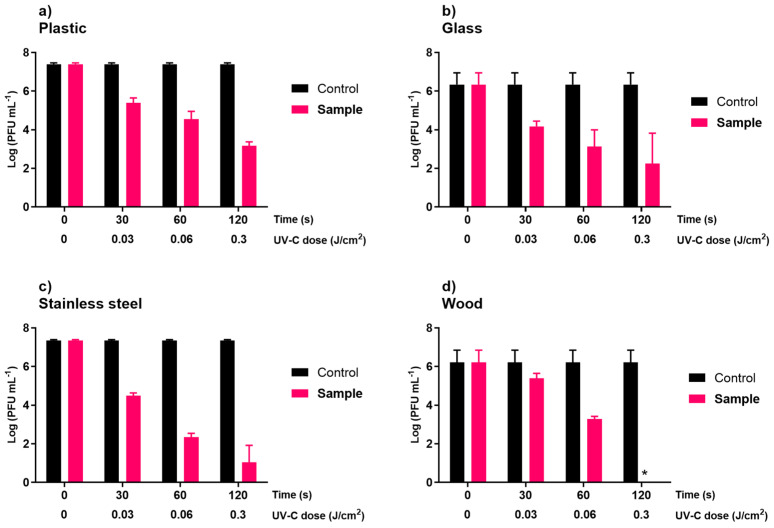
Inactivation of phage ϕ6 by the UV-C sterilizing chamber (2)2.4W on inanimate surfaces: plastic (**a**), glass (**b**), stainless steel (**c**), and wood (**d**). Limit of detection was about 2.3 log PFU/mL Values represent the mean of the three experiments and error bars represent the standard deviation. * below the methods’ detection limit.

**Figure 3:**
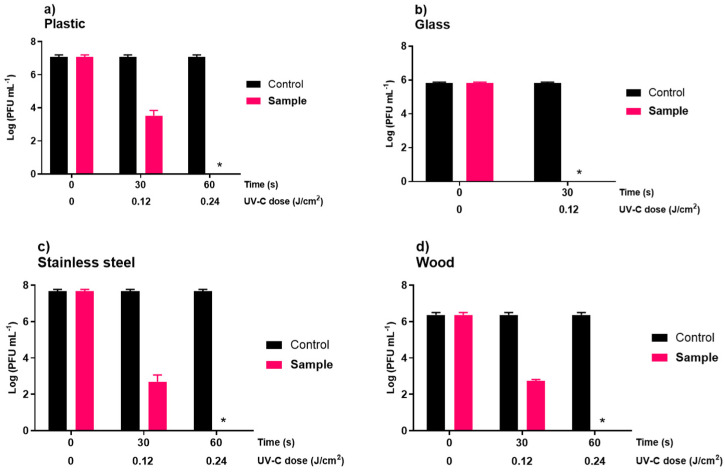
Inactivation of phage ϕ6 by the UV-C sterilizing chamber (8)5.5W on inanimate surfaces: plastic (**a**), glass (**b**), stainless steel (**c**), and wood (**d**). Limit of detection was about 2.3 log PFU/mL Values represent the mean of the three experiments and error bars represent the standard deviation. * below the methods’ detection limit.

In the phage inactivation assays, it was observed that the UV-C sterilizing chamber (8)5.5W system inactivated the phage faster than the UV-C sterilizing chamber (2)2.4W. In both irradiation systems, differences were observed in the viral inactivation rate between each inanimate surface tested (Figure 2 and Figure 3). In all experiments (Figure 2 and Figure 3), the controls remained stable throughout the assay.

In the UV-C sterilizing chamber (2)2.4W, the maximum viral inactivation was 4.1, 4.2, 6.3, and 6.2 log PFU/mL (ANOVA, *p* < 0.05) in plastic, glass, stainless steel, and wood, respectively, achieved after 120 s of irradiation (Figure 2). However, in plastic, after 30 and 60 s of irradiation, the viral inactivation was already 2.0 and 2.9 log PFU/mL (ANOVA, *p* < 0.05, Figure 2a). These results were similar (ANOVA, *p* > 0.05) to those observed in glass, with a 2.2 and 3.2 log PFU/mL decrease after 30 and 60 s of irradiation, respectively (Figure 2b). In general, the viral inactivation in stainless steel (decrease 2.9, 5.0, and 6.3 log PFU/mL after 30, 60, and 120 s of irradiation, respectively; Figure 2c) was higher than that observed in glass, plastic, and wood. In wood (Figure 2d), the maximum rate of inactivation was similar (ANOVA, *p* > 0.05) to that obtained for the stainless steel after 120 s of irradiation. However, after 30 and 60 s of irradiation, the viral inactivation for wood (decrease 0.8 and 2.9 log PFU/mL after 30 and 60 s, respectively) was lower (ANOVA, *p* < 0.05, Figure 2c,d) than that observed in stainless steel.

The UV-C sterilizing chamber (8)5.5W was able to inactivate phage ϕ6 below the detection limit of the method in all inanimate surfaces tested. In plastic, the detection limit of the method (inactivation of 7.1 log PFU/mL) was reached after 60 s (Figure 3a). However, the viral concentration decreased by approximately 3.6 log PFU/mL (ANOVA, *p* < 0.05, Figure 3a) after 30 s of irradiation. When the glass was disinfected with the UV-C light disinfection system, a reduction of approximately 6.0 log PFU/mL (ANOVA, *p* < 0.05, Figure 3b) was observed just after 30 s of irradiation, reaching the detection limit of the method. In stainless steel plates (Figure 3c), the inactivation reached the detection limit (7.7 log PFU/mL) after 60 s of irradiation (with 5.0 log PFU/mL decrease after just 30 s of irradiation). In wood (Figure 3d), a 3.7 log PFU/mL inactivation was achieved after 30 s, and the detection limit of the method was reached after 60 s of irradiation (6.4 log PFU/mL decrease).

#### 3.1.2. Personal Protective Equipment

**Figure 4:**
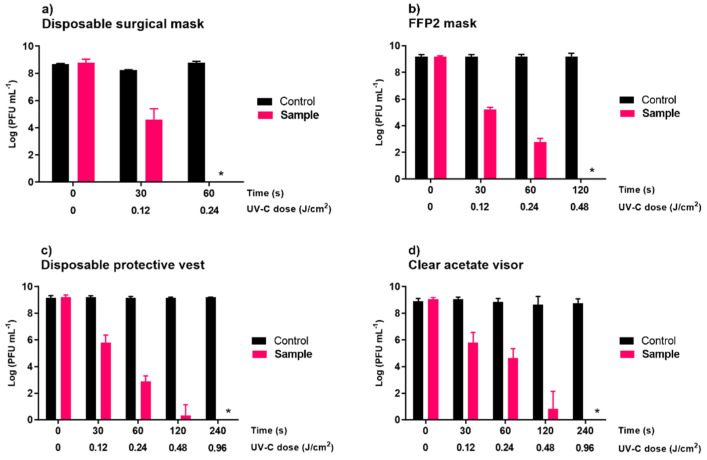
Inactivation of phage ϕ6 by the UV-C sterilizing chamber (8)5.5W on disposable surgical (**a**) and FFP2 (**b**) masks, disposable protective vest (**c**), and clear acetate visor (**d**). Limit of detection was about 2.3 log PFU/mL Values represent the mean of the three experiments and error bars represent the standard deviation. * below the methods’ detection limit.

In the UV-C sterilizing chamber (8)5.5W, viral inactivation was demonstrated to be material-dependent; the detection limit of the method was reached after 60, 120, 240, and 240 s of irradiation for disposable surgical masks, FFP2 masks, clear acetate visors, and disposable protective vests, respectively. In all assays (Figure 4), the controls remained constant throughout the experiment.

When the surgical masks were disinfected with the UV-C light disinfection system, a reduction of approximately 8.8 log PFU/mL (ANOVA, *p* < 0.05, Figure 4a) was observed after 60 s of irradiation, reaching the detection limit of the method. However, after 30 s of irradiation, the viral inactivation was already high (4.2 log PFU/mL).

In the FFP2 masks, a reduction of approximately 9.2 log PFU/mL (ANOVA, *p* < 0.05, Figure 4b) was observed after 120 s of irradiation, reaching the detection limit of the method. However, after 30 and 60 s of irradiation, the viral inactivation was already 4.0 and 6.4 log PFU/mL (ANOVA, *p* < 0.05, Figure 4b), respectively.

In the clear acetate visor and disposable protective vests, the detection limit of the method (with a 9.2 log PFU/mL reduction) was only reached after 240 s. After 30, 120, and 180 s of irradiation, the viral inactivation for clear acetate visor and disposable vests was similar (ANOVA, *p* > 0.05). However, after 60 s of irradiation, the viral inactivation for the disposable vest (decrease 6.3 log PFU/mL) was higher than that observed for the clear acetate visor (decrease 4.5 log PFU/mL).

## 4. Discussion

Some reports affirmed that the SARS-CoV-2 virus can remain on different surfaces for variable periods while maintaining its infective potential [5,6,18]. These studies highlight the potential importance of surface contamination for the dissemination of SARS-CoV-2 in the population. This fact emphasizes the importance of proper surface disinfection and its impact on potential infections. Furthermore, there is still little evidence that UV light may be effective for inactivating SARS-CoV-2 [7,32]. In this study, the results showed that the UV-C light was effective in inactivating phage ϕ6. It is important to note that the viral concentration used in this study was higher than reported by Kampf et al. (2020) in naturally contaminated surfaces. According to these authors, the SARS-CoV-2 concentrations per swab were 4.4–5.2 log_10_ in intensive care units and 2.8–4.0 log10 in general wards [9]. This demonstrates that UV-C systems can be effective against viral concentrations higher than those reported in the literature corresponding to naturally contaminated surfaces.

Hygiene practices for surfaces have led to several modifications in human habits and the development of innovative and efficient cleaning procedures [7]. In addition to chemicals, physical disinfection methods can be fast and less time-consuming thanks to the possibility of being carried out by automated instruments and applied on solid surfaces and aerosols [17]. In this study, both of the new UV-C systems reduced phage ϕ6 concentration in magnitudes, depending on the irradiance power between the UV-C systems and the UV dosage within the UV-C systems (Figure 2 and Figure 3). As the disinfection time increased, the effective reduction of phage ϕ6 of both systems increased. The results showed that the UV-C sterilizing chamber (8)5.5W (irradiance of 4.0 mW/cm^2^) system inactivated the phage ϕ6 faster than the UV-C sterilizing chamber (2)2.4W (irradiance of 1.0–2.5 mW/cm^2^) (Figure 2 and Figure 3). For the inanimate surfaces, the UV-C sterilizing chamber (8)5.5W was able to inactivate phage ϕ6 below the detection limit of the method after 30–60 s of irradiation (UV-C doses of 0.12–0.24 J/cm^2^) (Figure 3). In the UV-C sterilizing chamber (2)2.4W, the maximum viral inactivation was 4.1, 4.2, 6.3, and 6.2 log PFU/mL in plastic, glass, stainless steel, and wood, respectively, achieved after 120 s of irradiation (UV-C dose of 0.3 J/cm^2^) (Figure 2). Based on our results, UV-C irradiation of 4.0 mW/cm^2^ applied for 60 s (giving a UV-C dose of 0.24 J/cm^2^) is enough to reduce the viral titer below the detection limit of the method. Tseng and Li (2007) observed that a UV dose of 3.80 to 5.36 mJ/cm^2^ was required to efficiently reduce phage ϕ6 titer (90%) [51]. According to some studies, single-stranded nucleic acid viruses (such as SARS-CoV-2) are more susceptible to UV inactivation on surfaces than double-stranded nucleic acid viruses (such as phage ϕ6) [51,52]. Ma et al. (2021) evaluated the efficiency of five UV-C devices in the inactivation of three enveloped viruses (two coronaviruses, HCoV 229E and MHV; and phage ϕ6) and observed that phage ϕ6 was more resistant to UV radiation damage than the two coronaviruses [52]. In addition, phages are more resistant to UV-C radiation than other pathogenic viruses in the environment [51]. The shorter genome size of phages, compared to the animal viruses, leads to its higher UV resistance [23,53]. The nucleic acid sequence composition in the genome also affects the viral UV irradiation sensitivity in which adjacent pyrimidines are photoreactive under UV-C irradiation, forming pyrimidine dimers [1,18,19,52]. Thus, fewer adjacent pyrimidines in a phage’s genome may also contribute to its higher UV resistance. So, inactivation methods that are effective against phage ϕ6 will be also effective against the SARS-CoV-2. Therefore, viruses such as SARS-CoV-2 may be more susceptible to UV-C inactivation than the phage used in this study. However, further studies using different viruses and different viral concentrations are necessary to validate these results. It should be highlighted that only one phage was studied and the current results need to be further validated for other viruses, including the SARS-CoV-2.

The effectiveness of UV-C light also depends on the type of surface on which it is applied [18]. In the UV-C sterilizing chamber (2)2.4W, the maximum viral inactivation was 4.1, 4.2, 6.3, and 6.2 log PFU/mL in plastic, glass, stainless steel, and wood, respectively, achieved after 120 s of irradiation (UV-C dose of 0.3 J/cm^2^). The UV-C sterilizing chamber (8)5.5W was able to inactivate phage ϕ6 below the detection limit of the method after only 30 s in glass (UV-C dose of 0.12 J/cm^2^) and 60 s (UV-C dose of 0.24 J/cm^2^) in plastic, stainless steel, and wood. However, the viral concentration decreased by approximately 3.6, 5.0, and 3.7 log PFU/mL in plastic, stainless steel, and wood after 30 s of irradiation. Similar results were obtained by Gidari et al. (2021). These authors observed that plastic and stainless steel surfaces needed higher UV-C doses to achieve the target reduction [7]. The total inactivation of SARS-CoV-2 on glass was obtained with the lowest dose applied. Uneven and porous surfaces are considered problematic due to the lack of UV-C light penetration. UV-C light does not readily penetrate solid surfaces as light is absorbed or reflected by the substrate material. Decontamination efficacy decreases if the UV-C light cannot effectively penetrate shielded areas [51]. According to certain authors, plastic seems to be the most UV-C refractory material, followed by stainless steel and glass [7].

Due to the increased demand for PPE as a result of the current SARS-CoV-2 pandemic, decontamination and reuse of disposable surgical and FFP2 masks, disposable protective vests, and clear acetate visors may be required to ensure continued availability. Córdoba-Lánus et al. (2021) observed that PPE materials (gowns and masks) infected with a SARS-CoV-2 positive clinical sample retained their infectivity up to 5–7 days post-infection [54]. In another study, the authors observed that the SARS-CoV-2 maintained its infectiveness for 7 days post-infection in surgical masks [5]. Our results showed a reduction of approximately 8.8 and 9.2 log PFU/mL in disposable surgical and FFP2 masks, reaching the detection limit of the method, after 60 (UV-C dose of 0.24 J/cm^2^) and 120 s (UV-C dose of 0.48 J/cm^2^) of irradiation, respectively. The FFP2 masks contain multiple layers of filtration and breathing droplets may penetrate the inner layers. Fisher et al. (2011) demonstrated that the UV-C light was able to penetrate FFP masks, however, light transmittance ranges from 23–50% through the outer layer, depending on the FFP mask model [55]. In the clear acetate visor and disposable protective vests, the detection limit of the method (with a 9.1 log PFU/mL reduction) was only reached after 240 s (UV-C dose of 0.96 J/cm^2^). The ability of UV-C light to thoroughly sanitize PPE may vary based on its ability to penetrate the material.

In the future, it will be essential to understand the efficacy of UV-C light on SARS-CoV-2 using different viral concentrations, environmental conditions, and materials. Bianco et al. (2020) demonstrated that viral inhibition varied as a function of UV-C intensity and viral concentration [56]. Some studies have also shown that viral stability seems to be influenced by the characteristics of the different materials but also by environmental conditions such as temperature, pH, and humidity [5,12,13,14]. However, these factors were not considered in the current study.

## 5. Conclusions

UV-C irradiation can provide efficient, rapid, and sustainable sanitization procedures for different surfaces and PPE. Both UV-C systems were effective in inactivating phage ϕ6, but that effectiveness was material-dependent. Plastic, stainless steel, and wood surfaces (60 s) needed more time to achieve the viral inactivation to the detection limit of the method when compared with glass (30 s). For the disposable surgical masks and FFP2 masks, the viral inactivation was also more effective (60 and 120 s, respectively, to reach the detection limit of the method) than the inactivation in clear acetate visors and disposable protective vests (240 s to reach the detection limit of the method). Irradiance and irradiation period are important parameters to consider in the implementation of this technology as an important approach to fight the SARS-CoV-2 pandemic. Further studies are needed to validate these findings using SARS-CoV-2 at different concentrations, in different environmental conditions, and in different materials.

## Data Availability

Not applicable.
